# Supplementary material for: Sputum scarcity among adolescents and adults with presumptive tuberculosis: a systematic review and meta-analysis
Source: medRxiv. 2025 Nov 4:2025.11.02.25339326. Preprint. [Version 1] doi: 10.1101/2025.11.02.25339326 (PMC12637750; doi:10.1101/2025.11.02.25339326)
Supplement: Supplement 1 [file media-1.pdf]

Sputum scarcity among adolescents and adults with presumptive tuberculosis: a systematic review and meta-analysis

Supplementary Appendix

Table S1. Search Strategy

**PubMed**	
**P**	
1	"Mycobacterium tuberculosis"[MeSH Terms] OR "Tuberculosis"[MeSH Terms] OR "tuberculo*"[Title/Abstract] OR "TB"[Title/Abstract]
**I**	
2	"Sputum"[MeSH Terms] OR "sputum*"[Title/Abstract]
**C**	
3	"Urine"[MeSH Terms] OR "Feces"[MeSH Terms] OR "Blood"[MeSH Terms] OR "Serum"[MeSH Terms] OR "urin*"[Title/Abstract] OR "stool"[Title/Abstract] OR "fece*"[Title/Abstract] OR "Blood"[Title/Abstract] OR "breath*"[Title/Abstract] OR "serum*"[Title/Abstract]
**X**	
4	"Lipoarabinomannan"[Supplementary Concept] OR "Nucleic Acid Amplification Techniques"[MeSH Terms] OR "Lipoarabinomannan"[Title/Abstract] OR "LAM"[Title/Abstract] OR "AlereLAM"[Title/Abstract] OR "FujiLAM"[Title/Abstract] OR "LFLAM"[Title/Abstract] OR "TBLAM"[Title/Abstract] OR "Nucleic Acid Amplification"[Title/Abstract] OR "GeneXpert"[Title/Abstract] OR "xpert*"[Title/Abstract] OR "lamp loop"[Title/Abstract] OR "Truenat MTB"[Title/Abstract] OR "Culture"[Title/Abstract]
**Strings**<br>1-4 as in the tables above	
5	#2 OR #3
6	#1 AND #5 AND #4
7	#6 AND ("2010/01/01"[PDAT] : "3000/12/31"[PDAT])

**Embase**	
**P**	
1	'Mycobacterium tuberculosis'/exp OR 'tuberculosis'/exp OR Tuberculo*:ti,ab,kw OR TB:ti,ab,kw
**I**	
2	'sputum'/exp OR 'sputum examination'/exp OR sputum*:ti,ab,kw
**C**	
3	'urine'/exp OR 'feces'/exp OR 'blood'/exp OR 'serum'/exp OR 'feces analysis'/exp OR 'urinalysis'/exp OR urin*:ti,ab,kw OR stool:ti,ab,kw OR fece*:ti,ab,kw OR blood:ti,ab,kw OR breath*:ti,ab,kw OR serum*:ti,ab,kw
**X**	
4	'lipoarabinomannan'/exp OR 'nucleic acid amplification techniques'/exp OR Lipoarabinomannan:ti,ab,kw OR LAM:ti,ab,kw OR AlereLAM:ti,ab,kw OR FujiLAM:ti,ab,kw OR LFLAM:ti,ab,kw OR TBLAM:ti,ab,kw OR "Nucleic Acid Amplification":ti,ab,kw OR GeneXpert:ti,ab,kw OR Xpert*:ti,ab,kw OR "lamp loop":ti,ab,kw OR "Truenat MTB":ti,ab,kw OR Culture:ti,ab,kw
Strings	

1-4 as in tables above	
5	#2 or #3
6	#1 AND #5 AND #4
Publication year from 2010	
7	#6 AND [2010-2023]/py

**Cochrane Library**	
**P**	
1	[mh "Mycobacterium tuberculosis"] OR [mh "Tuberculosis"] OR Tuberculo*:ti,ab,kw OR TB:ti,ab,kw
**I**	
2	[mh "Sputum"] OR sputum*:ti,ab,kw
**C**	
3	[mh "Urine"] OR [mh "Feces"] OR [mh "Blood"] OR [mh "Serum"] OR urin*:ti,ab,kw OR stool:ti,ab,kw OR fece*:ti,ab,kw OR blood:ti,ab,kw OR breath*:ti,ab,kw OR serum*:ti,ab,kw
**X**	
4	[mh "Nucleic Acid Amplification Techniques"] OR Lipoarabinomannan:ti,ab,kw OR LAM:ti,ab,kw OR AlereLAM:ti,ab,kw OR FujiLAM:ti,ab,kw OR LFLAM:ti,ab,kw OR TBLAM:ti,ab,kw OR "Nucleic Acid Amplification":ti,ab,kw OR GeneXpert:ti,ab,kw OR Xpert*:ti,ab,kw OR "lamp loop":ti,ab,kw OR "Truenat MTB":ti,ab,kw OR Culture:ti,ab,kw
Strings<br>1-4 as in the tables above	
5	#2 OR #3
6	#1 AND #5 AND #4
Publication year from	
7	publication date from Jan 2010 to present<br>for CENTRAL applied in Endnote (729 > 684)

**Literatura Latino Americana en Ciencias de la Salud (LiLACS)**	
**P**	
1	Tuberculo* OR TB
**I / C**	
2	sputum* OR
3	urin* OR stool OR fece* OR blood OR breath* OR serum*
**X**	
4	Lipoarabinomannan OR LAM OR AlereLAM OR FujiLAM OR LFLAM OR TBLAM OR "Nucleic Acid Amplification" OR GeneXpert OR Xpert* OR "lamp loop" OR "Truenat MTB" OR Culture
Strings<br>1-4 as in the tables above in the fields Title, Abstract, Subject	
5	1 AND (2 OR 3) AND 4
	(tuberculo* OR tb) AND (sputum* OR urin* OR stool OR fece* OR blood OR breath* OR serum*) AND (lipoarabinomannan OR lam OR alerelam OR fujilam OR lflam OR tblam OR "Nucleic Acid Amplification" OR genexpert OR xpert* OR "lamp loop" OR "Truenat MTB" OR culture) AND (db:("WPRIM" OR "LILACS" OR "IBECS" OR "SES-SP" OR "AIM" OR "BINACIS" OR "LIPECS" OR "WHOLIS" OR "CUMED" OR "BDENF" OR "MedCarib" OR "HANSENIASE" OR "BIGG" OR "VETINDEX" OR "BBO")) AND (year_cluster:[2010 TO 2023])
Publication year from 2010	
6	Publication date from Jan 2010 to present

**Web of Science Core Collection**	
**P**	
1	Tuberculo* OR TB
**I**	
2	sputum*
**C**	
3	urin* OR stool OR fece* OR blood OR breath* OR serum*
**X**	
4	Lipoarabinomannan OR LAM OR AlereLAM OR FujiLAM OR LFLAM OR TBLAM OR "Nucleic Acid Amplification" OR GeneXpert OR Xpert* OR "lamp loop" OR "Truenat MTB" OR Culture
Strings<br>1-4 as in the tables above	
5	#2 OR #3
6	#1 AND #5 AND #4
Publication year from	
7	Publication year from 2010

**ClinicalTrial.gov**	
**P**	
1	tuberculosis OR TB
**I / C**	
2	Sputum OR
3	urine OR stool OR feces OR blood OR breath OR serum
**X**	
4	Lipoarabinomannan OR LAM OR AlereLAM OR FujiLAM OR LFLAM OR TBLAM OR "Nucleic Acid Amplification" OR GeneXpert OR Xpert OR "lamp loop" OR "Truenat MTB" OR Culture
Strings<br>1-4 as in the tables above	
5	1 AND (2 OR 3) AND 3
6	From 2010 (in Endnote applied)

**International Clinical Trials Registry Platform ICTRP (WHO Trials)**	
**P**	
1	tuberculosis OR TB
**I / C**	
2	sputum OR
3	urine OR stool OR feces OR blood OR breath OR serum
**X**	
4	Lipoarabinomannan OR LAM OR AlereLAM OR FujiLAM OR LFLAM OR TBLAM OR Nucleic Acid Amplification OR GeneXpert OR Xpert OR lamp loop OR Truenat MTB OR Culture
Strings (in advanced mode)	
5	1 AND (2 OR 3) AND 4

Table S2. Template for Risk of Bias assessment

**Domain**<br>**Signaling Question**	**Accepted values and answers**
**Domain 1: Patient Selection**<br>Could the selection of patients have introduced bias?	
1. Was a consecutive or random sample of patients enrolled?	Random → yes<br>Consecutive → yes<br>Convenience → no<br>NR → unsure
1. Was a case-control design avoided?	Cohort → yes<br>Cross-sectional → yes<br>Case-control → no<br>NR → unsure
2. Did the study avoid inappropriate exclusions? (e.g. previous TB, pregnancy, HIV)	Yes → yes<br>No → no<br>NR → unsure
Scoring:<br>Yes on ≥ 2 questions → Low<br>No on ≥ 2 questions → High<br>Unsure on ≥ 2 questions → Unsure	
**Domain 2: Applicability**<br>Is there a concern that the included participants do not match the review question?	
1. Were the study participants and setting described in detail, including information on screening and exclusions?	Yes → yes<br>No → no<br>NR → unsure
2. Were methods of sputum collection and exclusions described in detail?	Yes → yes<br>No → no<br>NR → unsure
3. Were results of sputum collection described in a standard, reliable way for all participants?	Yes → yes<br>No → no<br>NR → unsure
Scoring:<br>Yes on ≥ 2 questions → Low<br>No on ≥ 2 questions → High<br>Unsure on ≥ 2 questions → Unsure	

Table S3. Study definitions and outcomes

Term	Definition	Source
Self-expectorated sputum	Mucus coughed up from the respiratory tract by the patient without assistance	(1)
Sputum induction	Mucus coughed up from the respiratory tract after administration of nebulized saline	(2)
Sputum scarcity	The inability to provide an adequate sputum sample for TB testing	(3)
Spot samples	Sputum sample provided by self-expectoration during one clinical encounter or visit	(4)
Adequate samples	Samples determined to be of sufficient quality and volume required for testing. Definitions vary by study.	
Sputum quality	Based on the macroscopic appearance of the sample. Standard categories are salivary, mucoid, purulent, and blood-stained.	(5)
Sputum volume	Minium volume needed for diagnostic testing. May vary based on tests used.	(6, 7)

Table S4. Characteristics of included studies

Study ID	Design; DTA	Country (-ies)	Clinical setting	Healthcare level	N pts	TB prevalence (%)	HIV prevalence (%)	Previous TB (%)	Collection method	Number samples	Collection time	Sputum scarce, 95% (%)	Salivary (%)	RoB
Acharya, 2022 (8)	CSS; Yes	India	Mixed	Mixed	201	21.0	100	NR	NR	NR	NR	0.36 (0.30, 0.43)	NR	Low, Low
Adelman, 2015 (9)	CSS; no	Ethiopia	Outpatient	Tertiary	256	6.0	100	33.0	Self-exp	3	2 days	0.15 (0.11, 0.20)	NR	Low, Low
Ahsberg, 2023 (10)	Cohort; no	Ghana	Inpatient	Mixed	154	6.1	100	7.8	NR	1	Spot	0.27 (0.20, 0.35)	NR	Low, High
Alcantara 2012 (11)	CSS; no	Brazil	Outpatient	Mixed	265	18.9	NR	16.0	NR	2	2 days	0.06 (0.03, 0.09)	NR	Low, Unsure
Ali, 2021 (12)	Cohort; no	Pakistan	Outpatient	Tertiary	2896	0.93	NR	11.3	Self-exp	1	Spot	0.0 (0.0, 0.0)	NR	Low, Unsure
Alvarez, 2015 (13)	Cohort; yes	Canada	Mixed	Secondary	344	7.9	NR	NR	Induced if unable to exp	1-3	>2 days	0.0 (0.0, 0.01)	NR	Low, Low
Baghaei, 2011 (14)	CSS; yes	Iran	NR	Tertiary	809	61.0	0	0	NR	NR	NR	0.11 (0.08, 0.13)	NR	Low, High
Baik, 2020 (15)	C-C; no	Uganda	Outpatient	Primary	425	27.4	33.9	14.5	NR	NR	NR	0.04 (0.02, 0.06)	NR	Low, High
Balcells, 2012 (16)	CSS; yes	Chile	Mixed	Mixed	162	7.5	100	11.8	Self-exp	2	Spot	0.19 (0.13, 0.25)	NR	Low, Low
Balcha, 2014 (17)	Cohort; yes	Ethiopia	Outpatient	Primary	873	16.9	100	6.0	Self-exp	4	2 days	0.07 (0.05, 0.09)	NR	Low, High
Basir, 2019 (18)	CSS; no	Pakistan	Outpatient	Primary	153	22.8	NR	13.9	Self-exp	NR	NR	0.25 (0.18, 0.32)	NR	Low, High
Bassett, 2012 (19)	Cohort; no	South Africa	Outpatient	NR	951	15.0	100	26.0	Induced if unable to exp	1	Spot	0.0 (0.0, 0.0)	NR	Low, Low
Belay, 2015 (20)	CSS; no	Ethiopia	NR	Mixed	339	32.3	28.6	NR	NR	3	2 days	0.04 (0.02, 0.07)	NR	Low, Low
Benjamin 2019 (21)	CSS; yes	Brazil	Outpatient	Tertiary	205	24.6	100	23.1	Induced if unable to exp	2	NR	0.06 (0.03, 0.10)	NR	Low, Low
Berhanu 2018 (22)	CSS; yes	South Africa	Outpatient	Mixed	299	24.5	62	18.1	Self-exp	4	>2 days	0.03 (0.01, 0.06)	NR	Low, Low

Bigogo, 2018 (23)	CSS; no	Kenya	Outpatient	Mixed	7500	5.4	14.6	NR	Self-exp	1	Spot	0.83 (0.82, 0.84)	NR	Low, High
Bjerrum, 2015 (24)	Cohort; yes	Ghana	Mixed	Tertiary	495	11.7	100	6.0	Self-exp	2	>2 days	0.04 (0.03, 0.06)	NR	Low, Low
Bonnet, 2011 (25)	CSS; yes	Kenya	Outpatient	Primary	509	23.9	NR	20.6	Self-exp	4	>2 days	0.02 (0.01, 0.04)	3.7	Low, Low
Boum, 2013 (26)	CSS; yes	Uganda	Outpatient	Tertiary	891	23.7	58.6	2.6	NR	2	2 days	0.25 (0.22, 0.28)	0.3	Low, Low
Boyles, 2018 (27)	Cohort; yes	South Africa	Inpatient	Secondary	358	50.9	100	NR	Induced if unable to exp	3	NR	0.07 (0.05, 0.10)	NR	Low, Low
Boyles, 2020 (28)	Cohort; yes	South Africa	Outpatient	Primary	217	36.0	100	17.0	Induced if unable to exp	2	>2 days	0.0 (0.0, 0.02)	NR	Low, High
Burhan, 2022 (29)	CSS; no	Indonesia	Outpatient	Tertiary	490	69.8	5.2	42.0	Induced if unable to exp	NR	NR	0.04 (0.02, 0.06)	NR	Low, Low
Calder-wood, 2023 (30)	Cohort; yes	South Africa	Outpatient	Primary	1097	27.0	42.0	41.0	Induced if unable to exp	2	NR	0.08 (0.07, 0.10)	NR	Low, High
Carriquiry, 2012 (31)	CSS; yes	Peru	NR	Tertiary	152	34.4	100	25.0	Self-exp	2	2 days	0.11 (0.06, 0.17)	NR	Low, Low
Cattaman-chi, 2011 (32)	CSS; yes	Uganda	Inpatient	Tertiary	492	50.0	69.0	NR	NR	2	2 days	0.03 (0.02, 0.05)	21.4	Low, Low
Chaisson, 2015 (33)	CSS; yes	Vietnam	NR	Tertiary	332	28.5	0	NR	NR	2	NR	0.0 (0.0, 0.01)	NR	Low, High
Chawla, 2016 (34)	CSS; no	Malawi	Inpatient	Tertiary	658	9.1	30.4	NR	Self-exp	1	Spot	0.53 (0.49, 0.57)	NR	Low, Low
Chew, 2016 (35)	CSS; yes	Singapore	Inpatient	Tertiary	450	13.6	NR	NR	Induced if unable to exp	2	Spot	0.0 (0.0, 0.01)	NR	Low, High
Chilukutu, 2022 (36)	CSS; no	Zambia	Outpatient	Primary	771	12.4	43.1	19.3	NR	2	Spot	0.01 (0.00, 0.02)	NR	Low, High
Churchyard 2015 (37)	RCT; no	South Africa	Outpatient	Primary	4677	8.7	62	15.4	NR	1-2	NR	0.0 (0.0, 0.01)	NR	Low, High
Cowan, 2017 (38)	Cohort; no	United States of America	Inpatient	Tertiary	329	6.3	23.9	NR	Self-exp	1-3	2 days	0.03 (0.01, 0.06)	NR	Low, Low
Cox, 2014 (39)	RCT; no	South Africa	Outpatient	Primary	1985	21.5	48.6	37.5	NR	2	NR	0.02 (0.01, 0.03)	NR	Low, Low

Cuevas, 2011 (40)	CSS; yes	Ethiopia, Nepal, Nigeria, Yemen	NR	Mixed	6627	24.1	8.8	NR	Self-exp	3	2 days	0.04 (0.04, 0.05)	NR	Low, Low
Demelash, 2023 (41)	CSS; yes	Ethiopia	Outpatient	Tertiary	180	8.8	NR	NR	Self-exp	2	Spot	0.0 (0.0, 0.02)	NR	Unsure High
Der, 2021 (42)	CSS; no	Ghana	Outpatient	Secondary	236	3.2	10.8	3.8	Self-exp	1	Spot	0.20 (0.15, 0.26)	NR	Low, Low
Divala, 2023 (43)	RCT; no	Malawi	Outpatient	Primary	1583	6.3	14.9	6.3	NR	2	>2 days	0.18 (0.16, 0.20)	NR	Low, Low
Drain, 2014 (44)	CSS; yes	South Africa	Outpatient	Mixed	399	17.5	100	7.6	Induced if unable to exp	1	Spot	0.11 (0.08, 0.15)	NR	Low, Low
Dutschke, 2022 (45)	CSS; no	Guinea-Bissau	Outpatient	Tertiary	390	12.6	100	6.6	Self-exp	1	>2 days	0.48 (0.43, 0.53)	NR	Low, Low
El-Helbawy 2020 (46)	Cohort; yes	Egypt	NR	NR	452	24.7	NR	8.0	Induced if unable to exp	3	2 days	0.0 (0.0, 0.01)	88.4	Low, High
Fan, 2014 (47)	Cohort; yes	China	Inpatient	Tertiary	335	27.5	0	2.0	Self-exp	3	NR	0.24 (0.19, 0.29)	NR	Low, Low
Farr, 2019 (48)	CSS; no	Uganda	Outpatient	Primary	5330	8.5	47.9	0	NR	NR	NR	0.44 (0.43, 0.45)	NR	Low, High
Feasey, 2013 (49)	Cohort; yes	Malawi	Inpatient	Tertiary	104	43.0	100	0	Self-exp	3	2 days	0.19 (0.12, 0.28)	NR	Low, Low
Gammo, 2013 (50)	CSS; yes	Libya	NR	Tertiary	412	23.5	NR	NR	Self-exp	4	2 days	0.0 (0.0, 0.01)	NR	Low, Low
Gebreegziabiher, 2017 (51)	CSS; no	Ethiopia	Outpatient	NR	201	0.6	21.9	NR	Self-exp	3	2 days	0.13 (0.09, 0.19)	NR	Low, Low
Gounder, 2011 (52)	CSS; no	South Africa	Outpatient	Mixed	678	0.4	36.7	4.8	Self-exp	1	Spot	0.51 (0.47, 0.54)	NR	Low, Low
Grant, 2020 (53)	RCT; no	South Africa	Outpatient	Primary	1507	6.8	100	9.5	Self-exp	1	Spot	0.36 (0.33, 0.38)	NR	Low, High
Gray, 2016 (54)	CSS; yes	India, Uganda, Peru	NR	Mixed	1995	22.1	22.2	NR	NR	2	NR	0.03 (0.02, 0.04)	NR	Low, Low
Gupta-Wright 2018 (55)	RCT; yes	Malawi, South Africa	Inpatient	Tertiary	2574	11.5	100	25.0	Self-exp	1	Spot	0.43 (0.41, 0.45)	NR	Low, Low

Hanifa, 2012 (56)	Cohort; no	South Africa	Outpatient	Secondary	381	17.7	100	28.0	Self-exp	2	Spot	0.03 (0.01, 0.05)	NR	Low, Low
Hanifa, 2018 (57)	Cohort; no	South Africa	Outpatient	NR	367	7.0	100	24.0	Self-exp	1	Spot	0.36 (0.31, 0.41)	NR	Low, Low
Hanifa, 2019 (58)	Cohort; no	South Africa	Outpatient	NR	103	14.0	100	10.7	Induced if unable to exp	1	Spot	0.03 (0.01, 0.08)	NR	Low, Low
Huerga, 2017 (59)	Cohort; yes	Kenya	Mixed	Tertiary	474	56.7	100	24.7	Induced if unable to exp	2	2 days	0.23 (0.19, 0.27)	NR	Low, Low
Huerga, 2019 (60)	Cohort; no	Malawi, Mozambique	Outpatient	Mixed	456	24.9	100	NR	Self-exp	2	2 days	0.14 (0.11, 0.18)	NR	Low, Low
Huerga, 2020 (61)	Cohort; yes	Malawi	Outpatient	Mixed	485	14.1	100	NR	Self-exp	2	2 days	0.08 (0.06, 0.11)	NR	Low, Low
Huerga, 2021 (62)	CSS; yes	Malawi	Inpatient	Secondary	387	30.8	100	NR	Self-exp	2	>2 days	0.34 (0.30, 0.39)	NR	Low, Low
Huerga, 2023 (63)	CSS; yes	Uganda, Kenya, Mozambique, South Africa	Outpatient	Mixed	1031	9.4	100	NR	Induced if unable to exp	2	Spot	0.10 (0.08, 0.12)	NR	Low, Low
Jones-Lopez 2014 (64)	CSS; yes	Uganda	Inpatient	Tertiary	212	27.1	81.6	11.3	Self-exp	3	>2 days	0.0 (0.0, 0.02)	44.0	Low, Low
Kalema, 2012 (65)	CSS; no	Uganda	Inpatient	Tertiary	245	47.2	80.0	NR	Self-exp	2	Spot	0.08 (0.05, 0.12)	13.7	Low, Low
Kancheya, 2014 (66)	Cohort; no	Zambia	Outpatient	Primary	1422	1.5	17.0	2.9	Self-exp	2	Spot	0.11 (0.09, 0.12)	NR	Low, Low
Kasaro, 2020 (67)	CSS; yes	Zambia	Outpatient	Mixed	1350	15.4	100	NR	Self-exp	3	NR	0.20 (0.18, 0.22)	NR	High, Low
Kempker, 2019 (68)	CSS; no	Georgia	Outpatient	Tertiary	131	11.5	100	3.0	Self-exp	2	2 days	0.21 (0.15, 0.29)	NR	Low, Low
Khan, 2020 (69)	CSS; yes	Pakistan	Outpatient	Tertiary	2368	12.4	0.5	23.0	Induced if unable to exp	3	Spot	0.02 (0.02, 0.03)	NR	Low, Low
Kweza, 2018 (70)	CSS; no	South Africa	Outpatient	Primary	1255	5.4	17.7	14.4	Self-exp	1	Spot	0.28 (0.25, 0.30)	NR	Low, Low
Lawn, 2010 (71)	Cohort; no	South Africa	Outpatient	Primary	241	31.5	100	23.2	Induced if unable to exp	2	NR	0.0 (0.0, 0.02)	NR	Low, High

Lawn, 2012 (72)	CSS; yes	South Africa	Outpatient	Primary	602	17.3	100	26.5	Induced if unable to exp	2	Spot	0.10 (0.08, 0.13)	NR	Low, Low
Lawn, 2017 (73)	Cohort; yes	South Africa	Inpatient	Secondary	427	32.6	100	46.1	Induced if unable to exp	2	Spot	0.63 (0.58, 0.68)	NR	Low, Low
Lessells, 2017 (74)	RCT; no	South Africa	Outpatient	Primary	1281	12.9	92.5	39.0	Self-exp	2	Spot	0.04 (0.03, 0.05)	NR	Low, Low
Li, 2023 (75)	CSS; yes	China	Outpatient	Primary	396	25.5	NR	16.4	Induced if unable to exp	3	2 days	0.0 (0.0, 0.01)	NR	Low, High
Lodha, 2022 (76)	CSS; yes	India	Outpatient	Tertiary	114	43.9	1.8	NR	Induced if unable to exp	NR	NR	0.11 (0.06, 0.18)	NR	Low, Low
Lora, 2015 (77)	CSS; yes	Bolivia	Mixed	Tertiary	134	44.9	100	NR	Induced if unable to exp	3	>2 days	0.13 (0.08, 0.19)	NR	Low, Low
Mateyo, 2022 (78)	CSS; no	Zambia	Outpatient	Primary	771	13.0	67.0	19.0	NR	1	NR	0.01 (0.0, 0.02)	NR	Low, Low
Mathebula, 2020 (79)	CSS; no	Botswana	Outpatient	NR	1863	10.9	100	10.9	Self-exp	4	2 days	0.53 (0.51, 0.55)	29.3	Low, Low
Mbu, 2018 (80)	CSS; no	Cameroon	Outpatient	Tertiary	1149	13.9	100	NR	NR	2	2 days	0.18 (0.16, 0.21)	NR	Low, Low
Meyer, 2017 (81)	CSS; no	Uganda	Inpatient	Tertiary	3572	22.0	66.0	12.0	Induced if unable to exp	2	Spot	0.0 (0.0, 0.0)	16.0	Low, High
Miremba, 2012 (82)	CSS; yes	Uganda	Outpatient	Tertiary	231	50.7	35.7	8.1	Self-exp	3	2 days	0.01 (0.0, 0.03)	NR	Low, Low
Mtwangambate, 2014 (83)	Cohort; no	Tanzania	Mixed	Tertiary	121	5.8	59.	7.4	Self-exp	2	2 days	0.72 (0.63, 0.80)	NR	Low, Low
Munseri, 2011 (84)	Cohort; no	Tanzania	Inpatient	Tertiary	258	32.0	100	12.4	Self-exp	3	2 days	0.14 (0.10, 0.19)	NR	Low, Low
Mupfumi, 2014 (85)	RCT; yes	Zimbabwe	Outpatient	Tertiary	440	21.0	100	NR	Induced if unable to exp	2	Spot	0.04 (0.02, 0.06)	NR	Low, Low
Muyoyeta, 2021 (86)	CSS; yes	Zambia	Outpatient	Primary	157	22.5	46.0	21.2	Self-exp	1	Spot	0.0 (0.0, 0.02)	NR	Low, High
Nabeta, 2017 (87)	CSS; yes	Peru, Vietnam	Outpatient	Mixed	596	60.3	NR	NR	NR	2	Spot	0.04 (0.02, 0.06)	NR	Low, Low
Nakiyingi, 2014 (88)	CSS; yes	Uganda, South Africa	Mixed	Mixed	1013	36.8	100	19.0	Induced if unable to exp	2	Spot	0.02 (0.01, 0.03)	NR	Low, Low

Ngangue, 2022 (89)	CSS; yes	Cameroon	Outpatient	Secondary	1030	27.0	37.0	14.0	Self-exp	2	2 days	0.05 (0.04, 0.07)	NR	Low, Low
Nguyen, 2014 (90)	CSS; no	Vietnam	Inpatient	Tertiary	94	23.4	75.0	NR	Self-exp	NR	NR	0.35 (0.26, 0.46)	NR	Low, Low
Pandey, 2019 (91)	CSS; yes	India	NR	Mixed	290	30.0	0	NR	NR	2	2 days	0.18 (0.14, 0.23)	NR	High, High
Pant, 2022 (92)	CSS; no	Nepal	NR	Tertiary	104	9.6	NR	NR	NR	NR	NR	0.0 (0.0, 0.03)	NR	High, High
Penn-Nicholoson 2021 (93)	CSS; yes	Peru, India, Ethiopia, Papua New Guinea	Outpatient	Primary	1904	24.0	5.3	NR	Self-exp	4	2 days	0.05 (0.04, 0.06)	NR	Low, Low
Peter, 2012 (94)	CSS; yes	South Africa	Inpatient	Mixed	281	48.0	100	35.0	Self-exp	2	NR	0.26 (0.21, 0.32)	NR	Low, Low
Peter, 2016 (95)	RCT; yes	South Africa, Tanzania, Zambia, Zimbabwe	Inpatient	Secondary	2528	29.2	100	27.0	Induced if unable to exp	2	NR	0.07 (0.06, 0.08)	NR	Low, Low
Quinco, 2013 (96)	CSS; yes	Brazil	Mixed	Tertiary	508	19.4	60.4	3.0	NR	2	2 days	0.02 (0.01, 0.03)	NR	Low, Low
Rachow, 2022 (97)	Cohort yes	Romania	Outpatient	Tertiary	139	42.4	0.7	29.5	Self-exp	2	Spot	0.0 (0.0, 0.03)	NR	Low, Low
Reddy, 2010 (98)	CSS; no	Peru	NR	NR	471	6.2	100	14.3	Self-exp	4	>2 days	0.04 (0.02, 0.06)	NR	Low, Low
Reddy, 2017 (99)	CSS; yes	South Africa	Outpatient	Primary	717	23.3	NR	32.6	Self-exp	2	Spot	0.01 (0.0, 0.02)	NR	Low, Low
Reeve, 2023 (100)	CSS; yes	South Africa	Outpatient	Primary	897	12.0	100.0	14.0	Induced if unable to exp	3	Spot	0.02 (0.02, 0.04)	NR	Low, Low
Sander, 2019 (101)	CSS; no	Cameroon	Outpatient	Tertiary	1255	3.6	7.0	2.7	Self-exp	2	2 days	0.27 (0.24, 0.29)	NR	Low, High
Sani, 2020 (102)	CSS; no	Nigeria	Outpatient	Tertiary	216	19.9	0	NR	NR	3	>2 days	0.0 (0.0, 0.02)	NR	Low, High
Santoso, 2017 (103)	CSS; yes	Indonesia	NR	Tertiary	40	40.0	100	NR	Induced if unable to exp	1	Spot	0.0 (0.0, 0.08)	NR	Low, Low
Scott, 2011 (104)	Cohort; yes	South Africa	Outpatient	Primary	319	37.6	70.0	NR	Self-exp	3	>2 days	0.03 (0.01, 0.05)	NR	Low, Low

Seong, 2014 (105)	RCT; no	Republic of Korea	Outpatient	Tertiary	38	71.1	0	NR	Self-exp	3	>2 days	0.08 (0.02, 0.20)	NR	Low, Low
Shah, 2020 (106)	CSS; yes	South Africa, Uganda, India, Peru	Outpatient	NR	1086	32.0	47.0	NR	Self-exp	1	Spot	0.02 (0.01, 0.03)	NR	Low, Low
Shinu, 2013 (107)	CSS; yes	India	Outpatient	Tertiary	1184	31.5	0	NR	Self-exp	2	2 days	0.01 (0.00, 0.01)	5.3	Low, Low
Solari, 2019 (108)	CSS; no	Peru	Outpatient	Primary	237	5.1	0.9	8.6	Self-exp	1	Spot	0.12 (0.08, 0.17)	NR	Low, Low
Songkhla, 2019 (109)	CSS; yes	Thailand	Mixed	Tertiary	308	25.7	100	NR	Self-exp	1	NR	0.07 (0.05, 0.11)	NR	Low, Low
Spooner, 2022 (110)	CSS; yes	South Africa	Outpatient	Mixed	783	12.0	100	15.0	Induced if unable to exp	2	Spot	0.03 (0.02, 0.04)	NR	Low, Low
Theron, 2011 (7)	CSS; yes	South Africa	Outpatient	Primary	496	29.0	31.0	34.0	NR	2	Spot	0.38 (0.33, 0.42)	NR	Low, High
Vadwai, 2012 (111)	CSS; yes	India	NR	Tertiary	468	64.7	NR	NR	Self-exp	1	NR	0.04 (0.02, 0.06)	NR	Low, High
vanHoving, 2020 (112)	CSS; no	South Africa	Outpatient	Secondary	424	41.5	100	NR	Induced if unable to exp	2	2 days	0.44 (0.39, 0.49)	NR	Low, Low
vanLettow, 2015(113)	Cohort; no	Malawi	Outpatient	Mixed	348	15.0	55.0	21.0	NR	NR	NR	0.07 (0.05, 0.10)	NR	Low, High
Vijayageeth a, 2019 (114)	CSS; no	India	Outpatient	Tertiary	77	1.3	0.1	0.6	Self-exp	1	Spot	0.91 (0.82, 0.96)	NR	Low, Low
Wake, 2022 (115)	CSS; yes	South Africa	Mixed	Tertiary	181	17.0	100	12.4	Induced if unable to exp	NR	NR	0.50 (0.42, 0.57)	NR	High, High
Wang, 2016 (116)	CSS; yes	China	NR	NR	270	62.4	NR	NR	Self-exp	1	NR	0.04 (0.02, 0.07)	NR	Unsure High
Zu, 2019 (117)	CSS; no	China	NR	Tertiary	440	55.7	NR	NR	Self-exp	3	2 days	0.00 (0.00, 0.01)	NR	Low, Low
Yeong, 2020 (118)	CSS; yes	Australia	Inpatient	Tertiary	64	16.1	0	23.2	Self-exp	3	2 days	0.08 (0.03, 0.17)	NR	Low, Low

Yu, 2023 (119)	Cohort no	China	NR	Tertiary	158	51.3	0	46.9	Self-exp	NR	NR	0.20 (0.14, 0.27)	NR	Low, Low
Zhao, 2022 (120)	CSS; yes	China	NR	Tertiary	605	25.9	NR	NR	NR	NR	NR	0.47 (0.43, 0.51)	NR	Low, High

Legend: (abbreviations)
Design: CSS=cross-sectional study; RCT=randomized controlled trial; DTA=diagnostic test accuracy (yes, no)
Clinical setting: inpatient, outpatient, mixed inpatient and outpatient, not reported (NR)
Healthcare level: Primary, Secondary, Tertiary, mixed levels, not reported (NR)
N pts: number of participants attempting sputum collection
Collection method: self-expectorated, induced if unable to self-expectorate, not reported (NR)
Number of samples attempted: 1-2, more than 2, not reported (NR)
Time of sample collection: spot, 1-2 days, 2 days, >2 days, not reported (NR)
Risk of Bias (RoB): Patient selection, Applicability

Figure S1: Meta-analysis of sputum scarcity for collection of self-expectorated 1-2 spot sputum samples

Study	Method	No. of samples	Collect time	No. of patients	Proportion Sputum Scarce with 95% CI
Ali 2021	Exp	1	Spot	2896	0.00 [-0.00, 0.00]
Demelash 2023	Exp	2	Spot	180	0.00 [-0.00, 0.00]
Muyoyeta 2021	Exp	1	Spot	157	0.00 [-0.00, 0.00]
Rachow 2022	Exp	2	Spot	139	0.00 [-0.00, 0.00]
Meyer 2017	Exp	2	Spot	3572	0.01 [0.00, 0.01]
Reddy 2017	Exp	2	Spot	717	0.01 [0.00, 0.02]
Shah 2020	Exp	1	Spot	1086	0.02 [0.01, 0.03]
Hanifa 2012	Exp	2	Spot	381	0.03 [0.01, 0.05]
Lessells 2017	Exp	2	Spot	1281	0.04 [0.03, 0.05]
Spooner 2022	Exp	2	Spot	783	0.07 [0.05, 0.09]
Kalema 2012	Exp	2	Spot	245	0.08 [0.05, 0.12]
Kancheya 2014	Exp	2	Spot	1422	0.11 [0.09, 0.12]
Solari 2019	Exp	1	Spot	237	0.12 [0.08, 0.16]
Bassett 2012	Exp	1	Spot	951	0.18 [0.15, 0.20]
Balcells 2012	Exp	2	Spot	162	0.19 [0.13, 0.25]
Der 2021	Exp	1	Spot	236	0.20 [0.15, 0.25]
Huerga 2023	Exp	2	Spot	1031	0.27 [0.24, 0.30]
Kweza 2018	Exp	1	Spot	1255	0.28 [0.25, 0.30]
Benjamin 2019	Exp	1	Spot	199	0.31 [0.25, 0.38]
Grant 2020	Exp	1	Spot	1507	0.36 [0.33, 0.38]
Hanifa 2018	Exp	1	Spot	367	0.36 [0.31, 0.41]
Gupta-Wright 2018	Exp	1	Spot	2574	0.43 [0.41, 0.45]
Gounder 2011	Exp	1	Spot	678	0.51 [0.47, 0.54]
Chew 2016	Exp	2	Spot	450	0.53 [0.48, 0.58]
Chawla 2016	Exp	1	Spot	658	0.53 [0.50, 0.57]
Bigogo 2018	Exp	1	Spot	7500	0.83 [0.82, 0.84]
Vijayageetha 2019	Exp	1	Spot	77	0.91 [0.84, 0.97]
**Overall**					0.23 [0.14, 0.33]

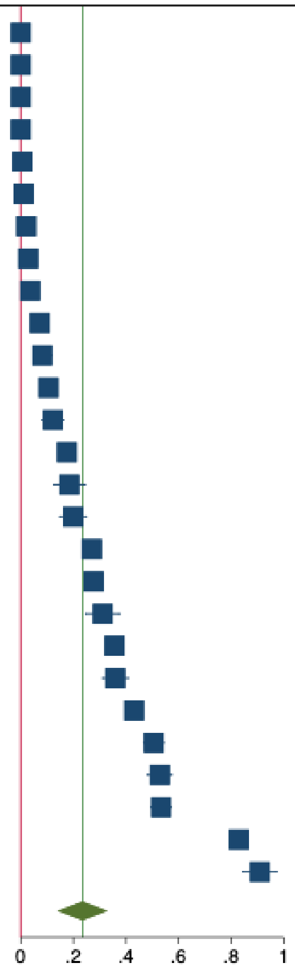

Figure S2: Meta-analysis of sputum scarcity in people living with HIV for collection of 1-2 self-expectorated spot sputum samples

Study	Method	No. of samples	Collect time	%PLHIV	No. of patients	Proportion Sputum Scarce with 95% CI
Hanifa 2012	Exp	2	Spot	100	381	0.03 [0.01, 0.05]
Spooner 2022	Exp	2	Spot	100	783	0.07 [0.05, 0.09]
Bassett 2012	Exp	1	Spot	100	951	0.18 [0.15, 0.20]
Balcells 2012	Exp	2	Spot	100	162	0.19 [0.13, 0.25]
Huerga 2023	Exp	2	Spot	100	1031	0.27 [0.24, 0.30]
Benjamin 2019	Exp	1	Spot	100	199	0.31 [0.25, 0.38]
Grant 2020	Exp	1	Spot	100	1507	0.36 [0.33, 0.38]
Hanifa 2018	Exp	1	Spot	100	367	0.36 [0.31, 0.41]
Gupta-Wright 2018	Exp	1	Spot	100	2574	0.43 [0.41, 0.45]
**Overall**						0.24 [0.15, 0.33]

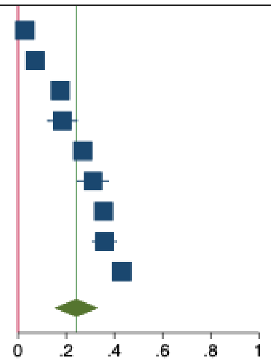

Figure S3: Meta-analysis of sputum scarcity for studies with mixed HIV status for collection of 1-2 self-expectorated spot sputum samples

Study	Method	No. of samples	Collect time	%PLHIV	No. of patients	Proportion Sputum Scarce with 95% CI
Muyoyeta 2021	Exp	1	Spot	46.0	157	0.00 [-0.00, 0.00]
Meyer 2017	Exp	2	Spot	66.0	3572	0.01 [0.00, 0.01]
Shah 2020	Exp	1	Spot	47.0	1086	0.02 [0.01, 0.03]
Lessells 2017	Exp	2	Spot	92.5	1281	0.04 [0.03, 0.05]
Kalema 2012	Exp	2	Spot	80.0	245	0.08 [0.05, 0.12]
Kancheya 2014	Exp	2	Spot	17.0	1422	0.11 [0.09, 0.12]
Der 2021	Exp	1	Spot	10.8	236	0.20 [0.15, 0.25]
Kweza 2018	Exp	1	Spot	17.7	1255	0.28 [0.25, 0.30]
Gounder 2011	Exp	1	Spot	36.7	678	0.51 [0.47, 0.54]
Chawla 2016	Exp	1	Spot	30.4	658	0.53 [0.50, 0.57]
Bigogo 2018	Exp	1	Spot	14.6	7500	0.83 [0.82, 0.84]
**Overall**						0.24 [0.07, 0.40]

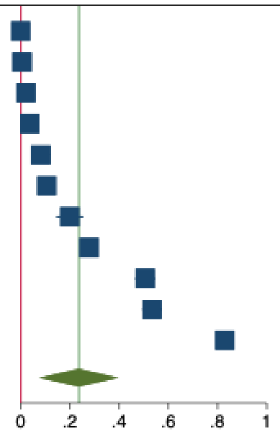

Figure S4. Meta-analysis of sputum scarcity in studies enrolling PLHIV before initiation of anti-retroviral therapy for collection of 1-2 self-expectorated sputum samples

Study	Method	No. of samples	Collect time	%PLHIV	No. of patients	Proportion Sputum Scarce with 95% CI
Hanifa 2012	Exp	2	Spot	100	381	0.03 [0.01, 0.05]
Spooner 2022	Exp	2	Spot	100	783	0.07 [0.05, 0.09]
Bassett 2012	Exp	1	Spot	100	951	0.18 [0.15, 0.20]
Grant 2020	Exp	1	Spot	100	1507	0.36 [0.33, 0.38]
Hanifa 2018	Exp	1	Spot	100	367	0.36 [0.31, 0.41]
**Overall**						0.20 [0.06, 0.33]

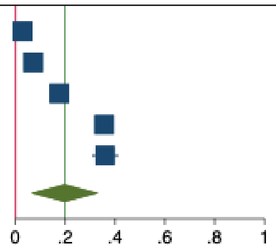

Figure S5: Meta-analysis of sputum scarcity for people not living with HIV for collection of any self-expectorated sputum samples

Study	Method	No. of samples	Collect time	%PLHIV	No. of patients	Proportion Sputum Scarce with 95% CI
Shinu 2013	Exp	2	1-2 days	0	1184	0.01 [0.00, 0.01]
Yeong 2020	Exp	3	1-2 days	0	64	0.08 [0.01, 0.14]
Seong 2014	Exp	3	>2 days	0	38	0.08 [-0.01, 0.16]
Yu 2023	Exp	.	.	0	158	0.20 [0.13, 0.26]
Fan 2014	Exp	3	.	0	335	0.24 [0.19, 0.28]
**Overall**						0.12 [0.03, 0.21]

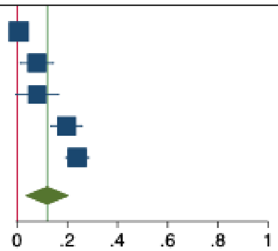

Figure S6. Meta-analysis of sputum scarcity in people living with HIV with advanced disease and/or inpatient settings for collection of 1-2 self-expectorated sputum samples

Study	Method	No. of samples	Collect time	%PLHIV	No. of patients	Proportion Sputum Scarce with 95% CI
Huerga 2019	Exp	2	1-2 days	100	456	0.14 [0.11, 0.17]
Benjamin 2019	Exp	1	Spot	100	199	0.31 [0.25, 0.38]
Huerga 2021	Exp	2	>2 days	100	387	0.34 [0.30, 0.39]
Hanifa 2018	Exp	1	Spot	100	367	0.36 [0.31, 0.41]
Gupta-Wright 2018	Exp	1	Spot	100	2574	0.43 [0.41, 0.45]
**Overall**						0.32 [0.22, 0.41]

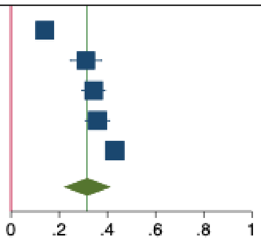

Figure S7. Meta-analysis of sputum scarcity in outpatient settings for collection of self-expectorated 1-2 spot sputum samples

Study	Method	No. of samples	Collect time	No. of patients	Proportion Sputum Scarce with 95% CI
Ali 2021	Exp	1	Spot	2896	0.00 [-0.00, 0.00]
Demelash 2023	Exp	2	Spot	180	0.00 [-0.00, 0.00]
Muyoyeta 2021	Exp	1	Spot	157	0.00 [-0.00, 0.00]
Rachow 2022	Exp	2	Spot	139	0.00 [-0.00, 0.00]
Reddy 2017	Exp	2	Spot	717	0.01 [0.00, 0.02]
Shah 2020	Exp	1	Spot	1086	0.02 [0.01, 0.03]
Hanifa 2012	Exp	2	Spot	381	0.03 [0.01, 0.05]
Lessells 2017	Exp	2	Spot	1281	0.04 [0.03, 0.05]
Spooner 2022	Exp	2	Spot	783	0.07 [0.05, 0.09]
Kancheya 2014	Exp	2	Spot	1422	0.11 [0.09, 0.12]
Solari 2019	Exp	1	Spot	237	0.12 [0.08, 0.16]
Bassett 2012	Exp	1	Spot	951	0.18 [0.15, 0.20]
Der 2021	Exp	1	Spot	236	0.20 [0.15, 0.25]
Huerga 2023	Exp	2	Spot	1031	0.27 [0.24, 0.30]
Kweza 2018	Exp	1	Spot	1255	0.28 [0.25, 0.30]
Reeve 2023	Exp	3	Spot	158	0.31 [0.24, 0.38]
Benjamin 2019	Exp	1	Spot	199	0.31 [0.25, 0.38]
Grant 2020	Exp	1	Spot	1507	0.36 [0.33, 0.38]
Hanifa 2018	Exp	1	Spot	367	0.36 [0.31, 0.41]
Gounder 2011	Exp	1	Spot	678	0.51 [0.47, 0.54]
Bigogo 2018	Exp	1	Spot	7500	0.83 [0.82, 0.84]
Vijayageetha 2019	Exp	1	Spot	77	0.91 [0.84, 0.97]
**Overall**					0.22 [0.11, 0.33]

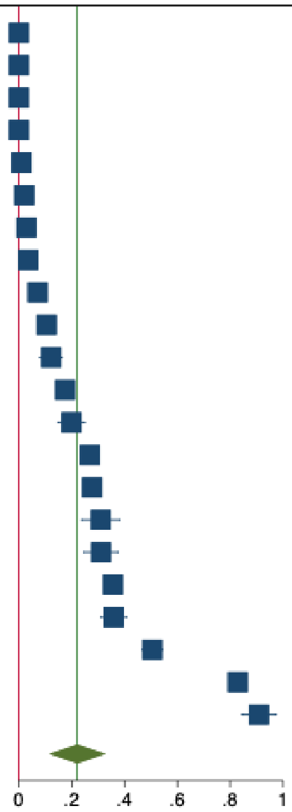

Figure S8: Meta-analysis of sputum scarcity for collection of induced 1-2 spot samples

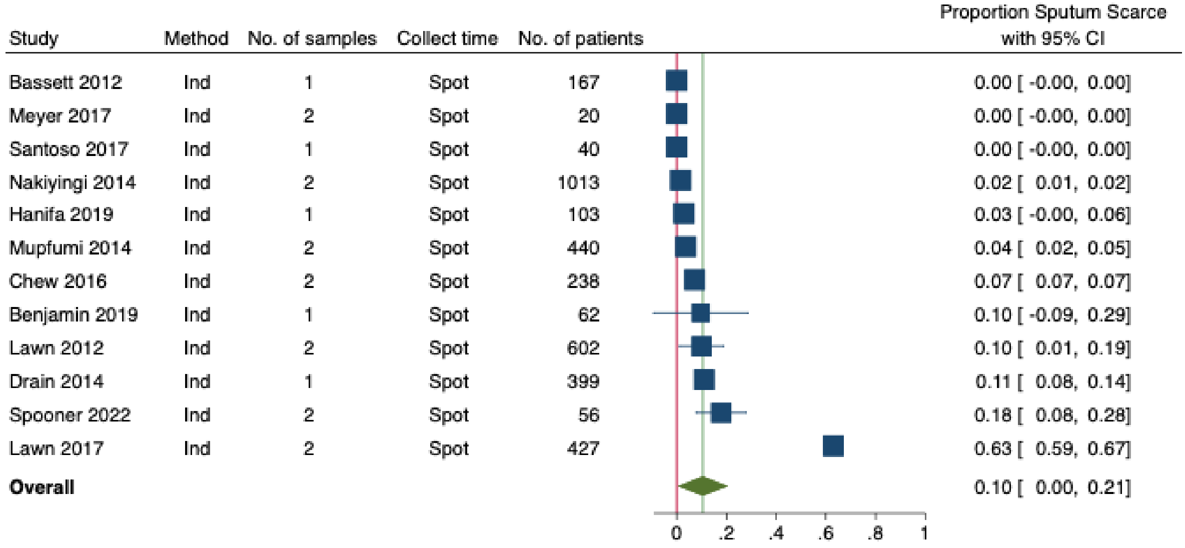

Figure S9. Meta-analysis of sputum scarcity for collection of self-expectorated 1-2 spot samples from studies in high TB-burden countries

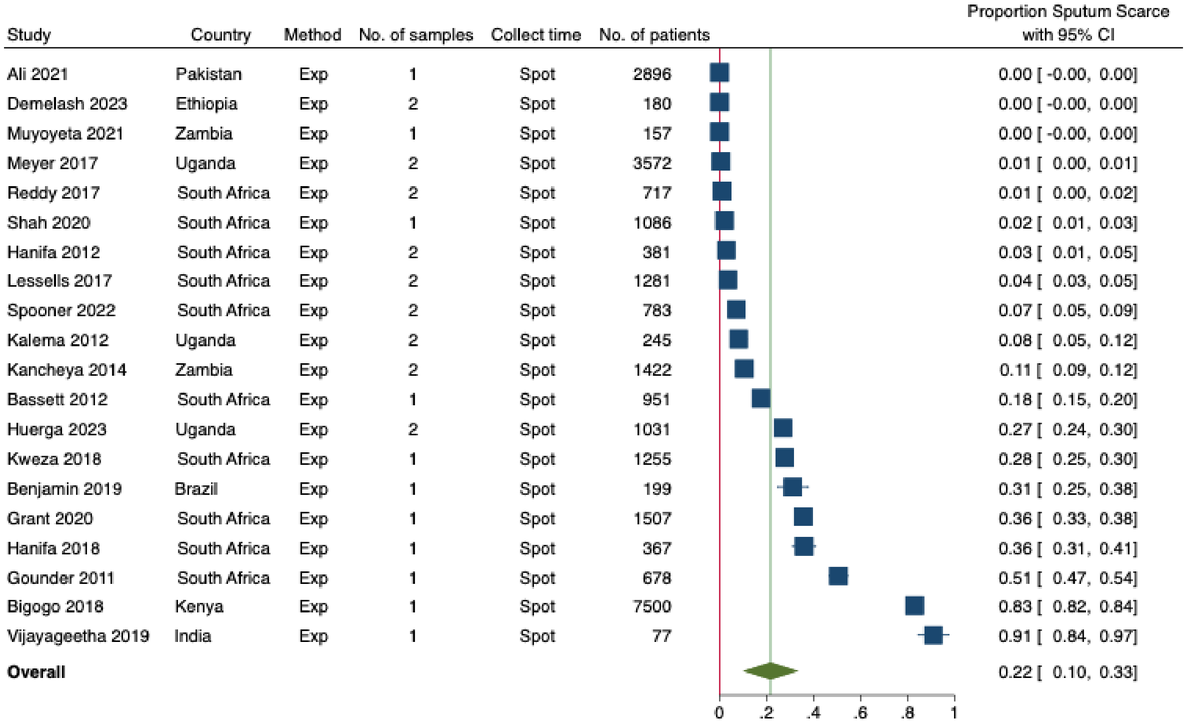

Figure S10. Meta-analysis of sputum scarcity for collection of self-expectorated 1-2 spot samples from studies in not high TB-burden countries

Study	Country	Method	No. of samples	Collect time	No. of patients	Proportion Sputum Scarce with 95% CI
Rachow 2022	Romania	Exp	2	Spot	139	0.00 [-0.00, 0.00]
Solari 2019	Peru	Exp	1	Spot	237	0.12 [0.08, 0.16]
Balcells 2012	Chile	Exp	2	Spot	162	0.19 [0.13, 0.25]
Der 2021	Ghana	Exp	1	Spot	236	0.20 [0.15, 0.25]
Gupta-Wright 2018	Malawi	Exp	1	Spot	2574	0.43 [0.41, 0.45]
Chew 2016	Singapore	Exp	2	Spot	450	0.53 [0.48, 0.58]
Chawla 2016	Malawi	Exp	1	Spot	658	0.53 [0.50, 0.57]
**Overall**						0.29 [0.13, 0.44]

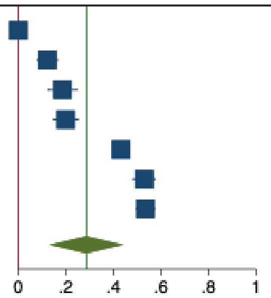

Figure S11. Meta-analysis of sputum scarcity for collection of self-expectorated 1-2 spot sputum samples; sensitivity analysis removing 'zero scarcity' studies

Study	Method	No. of samples	Collect time	No. of patients	Proportion Sputum Scarce with 95% CI
Reddy 2017	Exp	2	Spot	717	0.01 [0.00, 0.02]
Shah 2020	Exp	1	Spot	1086	0.02 [0.01, 0.03]
Hanifa 2012	Exp	2	Spot	381	0.03 [0.01, 0.05]
Lessells 2017	Exp	2	Spot	1281	0.04 [0.03, 0.05]
Spooner 2022	Exp	2	Spot	783	0.07 [0.05, 0.09]
Kalema 2012	Exp	2	Spot	245	0.08 [0.05, 0.12]
Kancheya 2014	Exp	2	Spot	1422	0.11 [0.09, 0.12]
Solari 2019	Exp	1	Spot	237	0.12 [0.08, 0.16]
Balcells 2012	Exp	2	Spot	162	0.19 [0.13, 0.25]
Der 2021	Exp	1	Spot	236	0.20 [0.15, 0.25]
Huerga 2023	Exp	2	Spot	1031	0.27 [0.24, 0.30]
Kweza 2018	Exp	1	Spot	1255	0.28 [0.25, 0.30]
Benjamin 2019	Exp	1	Spot	199	0.31 [0.25, 0.38]
Grant 2020	Exp	1	Spot	1507	0.36 [0.33, 0.38]
Hanifa 2018	Exp	1	Spot	367	0.36 [0.31, 0.41]
Gupta-Wright 2018	Exp	1	Spot	2574	0.43 [0.41, 0.45]
Gounder 2011	Exp	1	Spot	678	0.51 [0.47, 0.54]
Chawla 2016	Exp	1	Spot	658	0.53 [0.50, 0.57]
Bigogo 2018	Exp	1	Spot	7500	0.83 [0.82, 0.84]
Vijayageetha 2019	Exp	1	Spot	77	0.91 [0.84, 0.97]
**Overall**					0.28 [0.17, 0.39]

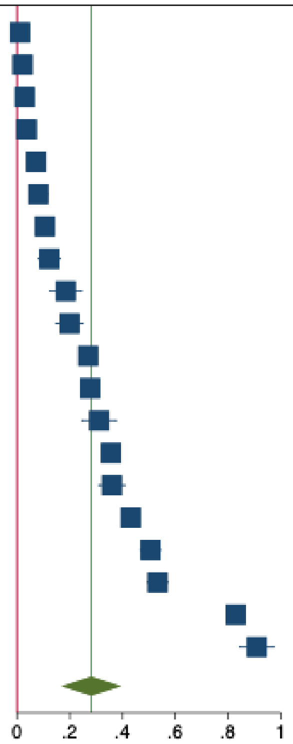

Table S5. Sensitivity analyses

Sub-group	Number of studies	Proportion Sputum Scarce (95%CI)
All patients, Self-expectorated, Spot, 1-2 samples (original estimate)	27	0.23 (0.14, 0.33)
All patients, Self-expectorated, 1-2 spot, removing studies with high Risk of Bias	21	0.22 (0.12, 0.32)
All patients, Self-expectorated, 1-2 spot, removing studies reporting sputum collection from all ("zero studies")	20	0.28 (0.17, 0.39)
Outpatients, Self-expectorated, 1-2 spot, removing studies reporting sputum collection from all ("zero studies")	16	0.28 (0.14, 0.41)

References

1. Farzan S. Clinical Methods: The History, Physical, and Laboratory Examinations.1990.
2. Peter JG, Theron G, Singh N, Singh A, Dheda K. Sputum induction to aid diagnosis of smear-negative or sputum-scarce tuberculosis in adults in HIV-endemic settings. European Respiratory Journal. 2014;43(1):185-94.
3. Vargas D, García L, Gilman RH, Evans C, Ticona E, Ñavincopa M, et al. Diagnosis of sputum-scarce HIV-associated pulmonary tuberculosis in Lima, Peru. The Lancet. 2005;365(9454):150-2.
4. Murphy ME, Phillips PPJ, Mendel CM, Bongard E, Bateson ALC, Hunt R, et al. Spot sputum samples are at least as good as early morning samples for identifying Mycobacterium tuberculosis. BMC Medicine. 2017;15(1):192.
5. Ho J, Marks GB, Fox GJ. The impact of sputum quality on tuberculosis diagnosis: a systematic review. Int J Tuberc Lung Dis. 2015;19(5):537-44.
6. Warren JR, Bhattacharya M, De Almeida KN, Trakas K, Peterson LR. A minimum 5.0 ml of sputum improves the sensitivity of acid-fast smear for Mycobacterium tuberculosis. Am J Respir Crit Care Med. 2000;161(5):1559-62.
7. Theron G, Peter J, van Zyl-Smit R, Mishra H, Streicher E, Murray S, et al. Evaluation of the Xpert MTB/RIF assay for the diagnosis of pulmonary tuberculosis in a high HIV prevalence setting. Am J Respir Crit Care Med. 2011;184(1):132-40.
8. Acharya S, Deshpande P, Asirvatham ES, Palkar A, Sarman CJ, Laxmeshwar C, et al. Utility of the lateral flow urine lipoarabinomannan tuberculosis assay in patients with advanced HIV disease at antiretroviral therapy centres in Mumbai, India. PLoS One. 2022;17(9):e0273970.
9. Adelman MW, Tsegaye M, Kempker RR, Alebachew T, Haile K, Tesfaye A, et al. Intensified tuberculosis case finding among HIV-infected persons using a WHO symptom screen and Xpert(®) MTB/RIF. Int J Tuberc Lung Dis. 2015;19(10):1197-203.
10. Åhsberg J, Bjerrum S, Ganu VJ, Kwashie A, Commey JO, Adusi-Poku Y, et al. The in-hospital tuberculosis diagnostic cascade and early clinical outcomes among people living with HIV before and during the COVID-19 pandemic - a prospective multisite cohort study from Ghana. Int J Infect Dis. 2023;128:290-300.
11. Alcântara CC, Kritski AL, Ferreira VG, Façanha MC, Pontes RS, Mota RS, et al. Factors associated with pulmonary tuberculosis among patients seeking medical attention at referral clinics for tuberculosis. J Bras Pneumol. 2012;38(5):622-9.
12. Ali RF, Siddiqi DA, Malik AA, Shah MT, Khan AJ, Hussain H, et al. Integrating tuberculosis screening into antenatal visits to improve tuberculosis diagnosis and care: Results from a pilot project in Pakistan. Int J Infect Dis. 2021;108:391-6.
13. Alvarez GG, Dyk DDV, Desjardlns M, Yasseen AS, 3rd, Aaron SD, Cameron DW, et al. The Feasibility, Accuracy, and Impact of Xpert MTB/RIF Testing in a Remote Aboriginal Community in Canada. Chest. 2015;148(3):767-73.
14. Baghaei P, Tabarsi P, Farnia P, Radaei AH, Kazempour M, Faghani YA, et al. Utility of Gastric Lavage for Diagnosis of Tuberculosis in Patients who are Unable to Expectorate Sputum. J Glob Infect Dis. 2011;3(4):339-43.
15. Baik Y, Rickman HM, Hanrahan CF, Mmolawa L, Kitonsa PJ, Sewelana T, et al. A clinical score for identifying active tuberculosis while awaiting microbiological results: Development and validation of a multivariable prediction model in sub-Saharan Africa. PLoS Med. 2020;17(11):e1003420.

16. Balcells ME, García P, Chanqueo L, Bahamondes L, Lasso M, Gallardo AM, et al. Rapid molecular detection of pulmonary tuberculosis in HIV-infected patients in Santiago, Chile. Int J Tuberc Lung Dis. 2012;16(10):1349-53.
17. Balcha TT, Sturegård E, Winqvist N, Skogmar S, Reepalu A, Jemal ZH, et al. Intensified tuberculosis case-finding in HIV-positive adults managed at Ethiopian health centers: diagnostic yield of Xpert MTB/RIF compared with smear microscopy and liquid culture. PLoS One. 2014;9(1):e85478.
18. Basir MS, Habib SS, Zaidi SMA, Khowaja S, Hussain H, Ferrand RA, et al. Operationalization of bi-directional screening for tuberculosis and diabetes in private sector healthcare clinics in Karachi, Pakistan. BMC Health Serv Res. 2019;19(1):147.
19. Bassett IV, Chetty S, Wang B, Mazibuko M, Giddy J, Lu Z, et al. Loss to follow-up and mortality among HIV-infected people co-infected with TB at ART initiation in Durban, South Africa. J Acquir Immune Defic Syndr. 2012;59(1):25-30.
20. Belay M, Bjune G, Abebe F. Prevalence of tuberculosis, HIV, and TB-HIV co-infection among pulmonary tuberculosis suspects in a predominantly pastoralist area, northeast Ethiopia. Glob Health Action. 2015;8:27949.
21. Benjamin A, Cavalcante SC, Jamal LF, Arakaki-Sanchez D, de Lima JN, Pilotto JH, et al. Accuracy of Determine TB-LAM Ag to detect TB in HIV infected patients associated with diagnostic methods used in Brazilian public health units. PLoS One. 2019;14(9):e0221038.
22. Berhanu RH, David A, da Silva P, Shearer K, Sanne I, Stevens W, et al. Performance of Xpert MTB/RIF, Xpert Ultra, and Abbott RealTime MTB for Diagnosis of Pulmonary Tuberculosis in a High-HIV-Burden Setting. J Clin Microbiol. 2018;56(12).
23. Bigogo G, Cain K, Nyole D, Masyongo G, Auko JA, Wamola N, et al. Tuberculosis case finding using population-based disease surveillance platforms in urban and rural Kenya. BMC Infect Dis. 2018;18(1):262.
24. Bjerrum S, Kenu E, Lartey M, Newman MJ, Addo KK, Andersen AB, et al. Diagnostic accuracy of the rapid urine lipoarabinomannan test for pulmonary tuberculosis among HIV-infected adults in Ghana-findings from the DETECT HIV-TB study. BMC Infect Dis. 2015;15:407.
25. Bonnet M, Gagnidze L, Githui W, Guérin PJ, Bonte L, Varaine F, et al. Performance of LED-based fluorescence microscopy to diagnose tuberculosis in a peripheral health centre in Nairobi. PLoS One. 2011;6(2):e17214.
26. Boum Y, 2nd, Orikiriza P, Rojas-Ponce G, Riera-Montes M, Atwine D, Nansumba M, et al. Use of colorimetric culture methods for detection of Mycobacterium tuberculosis complex isolates from sputum samples in resource-limited settings. J Clin Microbiol. 2013;51(7):2273-9.
27. Boyles TH, Griesel R, Stewart A, Mendelson M, Maartens G. Incremental yield and cost of urine Determine TB-LAM and sputum induction in seriously ill adults with HIV. Int J Infect Dis. 2018;75:67-73.
28. Boyles TH, Nduna M, Pitsi T, Scott L, Fox MP, Maartens G. A Clinical Prediction Score Including Trial of Antibiotics and C-Reactive Protein to Improve the Diagnosis of Tuberculosis in Ambulatory People With HIV. Open Forum Infect Dis. 2020;7(2):ofz543.
29. Burhan E, Karyana M, Karuniawati A, Kusmiati T, Wibisono BH, Handayani D, et al. Characteristics of Drug-sensitive and Drug-resistant Tuberculosis Cases among Adults at Tuberculosis Referral Hospitals in Indonesia. Am J Trop Med Hyg. 2022;107(5):984-91.

30. Calderwood CJ, Reeve BW, Mann T, Palmer Z, Nyawo G, Mishra H, et al. Clinical utility of C-reactive protein-based triage for presumptive pulmonary tuberculosis in South African adults. Journal of Infection. 2023;86(1):24-32.
31. Carriquiry G, Otero L, González-Lagos E, Zamudio C, Sánchez E, Nabeta P, et al. A diagnostic accuracy study of Xpert®MTB/RIF in HIV-positive patients with high clinical suspicion of pulmonary tuberculosis in Lima, Peru. PLoS One. 2012;7(9):e44626.
32. Cattamanchi A, Huang L, Worodria W, den Boon S, Kalema N, Katagira W, et al. Integrated strategies to optimize sputum smear microscopy: a prospective observational study. Am J Respir Crit Care Med. 2011;183(4):547-51.
33. Chaisson LH, Reber C, Phan H, Switz N, Nilsson LM, Myers F, et al. Evaluation of mobile digital light-emitting diode fluorescence microscopy in Hanoi, Viet Nam. Int J Tuberc Lung Dis. 2015;19(9):1068-72.
34. Chawla KS, Kanyama C, Mbewe A, Matoga M, Hoffman I, Ngoma J, et al. Policy to practice: impact of GeneXpert MTB/RIF implementation on the TB spectrum of care in Lilongwe, Malawi. Trans R Soc Trop Med Hyg. 2016;110(5):305-11.
35. Chew MY, Ng J, Cai HM, Lim TG, Lim TK. The clinical utility of Xpert® MTB/RIF testing in induced sputum. Int J Tuberc Lung Dis. 2016;20(12):1668-70.
36. Chilukutu L, Mwanza W, Kerkhoff AD, Somwe P, Kagujje M, Muyoyeta M. Prevalence and interpretation of Xpert(®) Ultra trace results among presumptive TB patients. Public Health Action. 2022;12(1):28-33.
37. Churchyard GJ, Stevens WS, Mametja LD, McCarthy KM, Chihota V, Nicol MP, et al. Xpert MTB/RIF versus sputum microscopy as the initial diagnostic test for tuberculosis: a cluster-randomised trial embedded in South African roll-out of Xpert MTB/RIF. Lancet Glob Health. 2015;3(8):e450-e7.
38. Cowan JF, Chandler AS, Kracen E, Park DR, Wallis CK, Liu E, et al. Clinical Impact and Cost-effectiveness of Xpert MTB/RIF Testing in Hospitalized Patients With Presumptive Pulmonary Tuberculosis in the United States. Clin Infect Dis. 2017;64(4):482-9.
39. Cox HS, Mbhele S, Mohess N, Whitelaw A, Muller O, Zemanay W, et al. Impact of Xpert MTB/RIF for TB diagnosis in a primary care clinic with high TB and HIV prevalence in South Africa: a pragmatic randomised trial. PLoS Med. 2014;11(11):e1001760.
40. Cuevas LE, Yassin MA, Al-Sonboli N, Lawson L, Arbide I, Al-Aghbari N, et al. A multi-country non-inferiority cluster randomized trial of frontloaded smear microscopy for the diagnosis of pulmonary tuberculosis. PLoS Med. 2011;8(7):e1000443.
41. Demelash M, Nibret E, Mnichil Z. Comparison of Ziehl Neelsen microscopy and geneXpert for the diagnosis of pulmonary tuberculosis among pulmonary tuberculosis-suspected patients in northwest Ethiopia. Trop Doct. 2023;53(3):362-5.
42. Der JB, Grint DJ, Narh CT, Bonsu F, Grant AD. Missed opportunities for tuberculosis investigation in a municipal hospital in Ghana: evidence from patient exit interviews. Trans R Soc Trop Med Hyg. 2021;115(1):43-50.
43. Divala TH, Corbett EL, Kandulu C, Moyo B, MacPherson P, Nliwasa M, et al. Trial-of-antibiotics to assist tuberculosis diagnosis in symptomatic adults in Malawi (ACT-TB study): a randomised controlled trial. Lancet Glob Health. 2023;11(4):e556-e65.
44. Drain PK, Losina E, Coleman SM, Giddy J, Ross D, Katz JN, et al. Diagnostic accuracy of a point-of-care urine test for tuberculosis screening among newly-diagnosed HIV-infected adults: a prospective, clinic-based study. BMC Infect Dis. 2014;14:110.

45. Dutschke A, Steiniche D, Jespersen S, Nanque JP, Medina C, Hønge BL, et al. Xpert MTB/RIF on urine samples to increase diagnosis of TB in people living with HIV in Guinea-Bissau. Int J Infect Dis. 2022;124 Suppl 1:S63-s8.
46. El-Helbawy RH, Abdel Tawab AM. GeneXpert Mycobacterial tuberculosis/rifampicin: predictors of successful performance. The Egyptian Journal of Chest Diseases and Tuberculosis. 2020;69(1).
47. Fan L, Zhang Q, Cheng L, Liu Z, Ji X, Cui Z, et al. Clinical diagnostic performance of the simultaneous amplification and testing methods for detection of the Mycobacterium tuberculosis complex for smear-negative or sputum-scarce pulmonary tuberculosis in China. Chin Med J (Engl). 2014;127(10):1863-7.
48. Farr K, Nalugwa T, Ojok C, Nantale M, Nabwire S, Oyuku D, et al. Quality of care for patients evaluated for tuberculosis in the context of Xpert MTB/RIF scale-up. J Clin Tuberc Other Mycobact Dis. 2019;15:100099.
49. Feasey NA, Banada PP, Howson W, Sloan DJ, Mdolo A, Boehme C, et al. Evaluation of Xpert MTB/RIF for detection of tuberculosis from blood samples of HIV-infected adults confirms Mycobacterium tuberculosis bacteremia as an indicator of poor prognosis. J Clin Microbiol. 2013;51(7):2311-6.
50. Gammo M, Lamaric W, Hadida M, Abuazza A, Askar NA, Yassin MA, et al. Front-loaded smear microscopy for the diagnosis of pulmonary TB in Tripoli, Libya. Trans R Soc Trop Med Hyg. 2013;107(2):137-9.
51. Gebreegziabiher D, Adane K, Abebe M. A survey on undiagnosed active pulmonary tuberculosis among pregnant mothers in mekelle and surrounding Districts in Tigray, Ethiopia. Int J Mycobacteriol. 2017;6(1):43-6.
52. Gounder CR, Wada NI, Kensler C, Violari A, McIntyre J, Chaisson RE, et al. Active tuberculosis case-finding among pregnant women presenting to antenatal clinics in Soweto, South Africa. J Acquir Immune Defic Syndr. 2011;57(4):e77-84.
53. Grant AD, Charalambous S, Tlali M, Karat AS, Dorman SE, Hoffmann CJ, et al. Algorithm-guided empirical tuberculosis treatment for people with advanced HIV (TB Fast Track): an open-label, cluster-randomised trial. Lancet HIV. 2020;7(1):e27-e37.
54. Gray CM, Katamba A, Narang P, Giraldo J, Zamudio C, Joloba M, et al. Feasibility and operational performance of tuberculosis detection by loop-mediated isothermal amplification platform in decentralized settings: results from a multicenter study. Journal of clinical microbiology. 2016;54(8):1984-91.
55. Gupta-Wright A, Corbett EL, van Oosterhout JJ, Wilson D, Grint D, Alufandika-Moyo M, et al. Rapid urine-based screening for tuberculosis in HIV-positive patients admitted to hospital in Africa (STAMP): a pragmatic, multicentre, parallel-group, double-blind, randomised controlled trial. Lancet. 2018;392(10144):292-301.
56. Hanifa Y, Fielding KL, Charalambous S, Variava E, Luke B, Churchyard GJ, et al. Tuberculosis among adults starting antiretroviral therapy in South Africa: the need for routine case finding. Int J Tuberc Lung Dis. 2012;16(9):1252-9.
57. Hanifa Y, Fielding KL, Chihota VN, Adonis L, Charalambous S, Foster N, et al. The utility of repeat Xpert MTB/RIF testing to diagnose tuberculosis in HIV-positive adults with initial negative result. Gates Open Res. 2018;2:22.
58. Hanifa Y, Silva ST, Karstaedt A, Sahid F, Charalambous S, Chihota VN, et al. What causes symptoms suggestive of tuberculosis in HIV-positive people with negative initial investigations? International journal of tuberculosis and lung disease. 2019;23(2):157-+.

59. Huerga H, Ferlazzo G, Bevilacqua P, Kirubi B, Ardizzoni E, Wanjala S, et al. Incremental Yield of Including Determine-TB LAM Assay in Diagnostic Algorithms for Hospitalized and Ambulatory HIV-Positive Patients in Kenya. PLoS One. 2017;12(1):e0170976.
60. Huerga H, Mathabire Rucker SC, Cossa L, Bastard M, Amoros I, Manhiça I, et al. Diagnostic value of the urine lipoarabinomannan assay in HIV-positive, ambulatory patients with CD4 below 200 cells/µl in 2 low-resource settings: A prospective observational study. PLoS Med. 2019;16(4):e1002792.
61. Huerga H, Rucker SCM, Bastard M, Dimba A, Kamba C, Amoros I, et al. Should Urine-LAM Tests Be Used in TB Symptomatic HIV-Positive Patients When No CD4 Count Is Available? A Prospective Observational Cohort Study From Malawi. J Acquir Immune Defic Syndr. 2020;83(1):24-30.
62. Huerga H, Mathabire Rucker SC, Bastard M, Mpunga J, Amoros Quiles I, Kabaghe C, et al. Urine Lipoarabinomannan Testing for All HIV Patients Hospitalized in Medical Wards Identifies a Large Proportion of Patients With Tuberculosis at Risk of Death. Open Forum Infect Dis. 2021;8(2):ofaa639.
63. Huerga H, Bastard M, Lubega AV, Akinyi M, Antabak NT, Ohler L, et al. Novel FujiLAM assay to detect tuberculosis in HIV-positive ambulatory patients in four African countries: a diagnostic accuracy study. Lancet Glob Health. 2023;11(1):e126-e35.
64. Jones-López E, Manabe YC, Palaci M, Kayiza C, Armstrong D, Nakiyingi L, et al. Prospective cross-sectional evaluation of the small membrane filtration method for diagnosis of pulmonary tuberculosis. J Clin Microbiol. 2014;52(7):2513-20.
65. Kalema N, Boon SD, Cattamanchi A, Davis JL, Andama A, Katagira W, et al. Oral antimicrobial rinse to reduce mycobacterial culture contamination among tuberculosis suspects in Uganda: a prospective study. PLoS One. 2012;7(7):e38888.
66. Kancheya N, Luhanga D, Harris JB, Morse J, Kapata N, Bweupe M, et al. Integrating active tuberculosis case finding in antenatal services in Zambia. Int J Tuberc Lung Dis. 2014;18(12):1466-72.
67. Kasaro MP, Chilyabanyama ON, Shah NS, Muluka B, Kapata N, Krüüner A, et al. Performance of Xpert(®) MTB/RIF and Determine™ TB-LAM Ag in HIV-infected adults in peri-urban sites in Zambia. Public Health Action. 2020;10(4):134-40.
68. Kempker RR, Chkhartishvili N, Kinkladze I, Schechter MC, Harrington K, Rukhadze N, et al. High Yield of Active Tuberculosis Case Finding Among HIV-Infected Patients Using Xpert MTB/RIF Testing. Open Forum Infect Dis. 2019;6(6):ofz233.
69. Khan FA, Majidulla A, Tavaziva G, Nazish A, Abidi SK, Benedetti A, et al. Chest x-ray analysis with deep learning-based software as a triage test for pulmonary tuberculosis: a prospective study of diagnostic accuracy for culture-confirmed disease. Lancet Digit Health. 2020;2(11):e573-e81.
70. Kweza PF, Van Schalkwyk C, Abraham N, Uys M, Claassens MM, Medina-Marino A. Estimating the magnitude of pulmonary tuberculosis patients missed by primary health care clinics in South Africa. Int J Tuberc Lung Dis. 2018;22(3):264-72.
71. Lawn SD, Kranzer K, Edwards DJ, McNally M, Bekker LG, Wood R. Tuberculosis during the first year of antiretroviral therapy in a South African cohort using an intensive pretreatment screening strategy. Aids. 2010;24(9):1323-8.
72. Lawn SD, Kerkhoff AD, Vogt M, Wood R. Diagnostic accuracy of a low-cost, urine antigen, point-of-care screening assay for HIV-associated pulmonary tuberculosis before antiretroviral therapy: a descriptive study. Lancet Infect Dis. 2012;12(3):201-9.

73. Lawn SD, Kerkhoff AD, Burton R, Schutz C, Boulle A, Vogt M, et al. Diagnostic accuracy, incremental yield and prognostic value of Determine TB-LAM for routine diagnostic testing for tuberculosis in HIV-infected patients requiring acute hospital admission in South Africa: a prospective cohort. BMC Med. 2017;15(1):67.
74. Lessells RJ, Cooke GS, McGrath N, Nicol MP, Newell ML, Godfrey-Faussett P. Impact of Point-of-Care Xpert MTB/RIF on Tuberculosis Treatment Initiation. A Cluster-randomized Trial. Am J Respir Crit Care Med. 2017;196(7):901-10.
75. Li M, Qiu Y, Guo M, Qu R, Tian F, Wang G, et al. Comparison of Xpert MTB/RIF Ultra with Xpert MTB/RIF for the detection of Mycobacterium tuberculosis and rifampicin resistance in a primary-level clinic in rural China. Tuberculosis (Edinb). 2023;142:102397.
76. Lodha L, Mudliar SR, Singh J, Maurya A, Khurana AK, Khadanga S, et al. Diagnostic Performance of Multiplex PCR for Detection of Mycobacterium tuberculosis Complex in Presumptive Pulmonary Tuberculosis Patients and Its Utility in Smear Negative Specimens. J Lab Physicians. 2022;14(4):403-11.
77. Lora MH, Reimer-McAtee MJ, Gilman RH, Lozano D, Saravia R, Pajuelo M, et al. Evaluation of Microscopic Observation Drug Susceptibility (MODS) and the string test for rapid diagnosis of pulmonary tuberculosis in HIV/AIDS patients in Bolivia. BMC Infect Dis. 2015;15:222.
78. Mateyo K, Kerkhoff AD, Dunn I, Nteeni MS, Muyoyeta M. Clinical and radiographic characteristics of presumptive tuberculosis patients previously treated for tuberculosis in Zambia. PLoS One. 2022;17(1):e0263116.
79. Mathebula U, Emerson C, Agizew T, Pals S, Boyd R, Mathoma A, et al. Improving sputum collection processes to increase tuberculosis case finding among HIV-positive persons in Botswana. Public Health Action. 2020;10(1):11-6.
80. Mbu ET, Sauter F, Zoufaly A, Bronsvoort BMC, Morgan KL, Noeske J, et al. Tuberculosis in people newly diagnosed with HIV at a large HIV care and treatment center in Northwest Cameroon: Burden, comparative screening and diagnostic yields, and patient outcomes. PLoS One. 2018;13(6):e0199634.
81. Meyer AJ, Atuheire C, Worodria W, Kizito S, Katamba A, Sanyu I, et al. Sputum quality and diagnostic performance of GeneXpert MTB/RIF among smear-negative adults with presumed tuberculosis in Uganda. PLoS One. 2017;12(7):e0180572.
82. Miremba P, Kalyango JN, Worodria W, Mugerwa H, Nakakawa E, Asiimwe BB. Performance of frontloading for smear microscopy in the diagnosis of pulmonary tuberculosis: a cross-sectional study at a referral hospital in Uganda. PLoS One. 2012;7(10):e48531.
83. Mtwangambate G, Kalluvya SE, Kidenya BR, Kabangila R, Downs JA, Smart LR, et al. 'Cough-triggered' tuberculosis screening among adults with diabetes in Tanzania. Diabet Med. 2014;31(5):600-5.
84. Munseri PJ, Talbot EA, Bakari M, Matee M, Teixeira JP, von Reyn CF. The bacteraemia of disseminated tuberculosis among HIV-infected patients with prolonged fever in Tanzania. Scand J Infect Dis. 2011;43(9):696-701.
85. Mupfumi L, Makamure B, Chirehwa M, Sagonda T, Zinyowera S, Mason P, et al. Impact of Xpert MTB/RIF on Antiretroviral Therapy-Associated Tuberculosis and Mortality: A Pragmatic Randomized Controlled Trial. Open Forum Infect Dis. 2014;1(1):ofu038.
86. Muyoyeta M, Kerkhoff AD, Chilukutu L, Moreau E, Schumacher SG, Ruhwald M. Diagnostic accuracy of a novel point-of-care urine lipoarabinomannan assay for the

detection of tuberculosis among adult outpatients in Zambia: a prospective cross-sectional study. Eur Respir J. 2021;58(5).
87. Nabeta P, Havumaki J, Ha DT, Caceres T, Hang PT, Collantes J, et al. Feasibility of the TBDx automated digital microscopy system for the diagnosis of pulmonary tuberculosis. PLoS One. 2017;12(3):e0173092.
88. Nakiyingi L, Moodley VM, Manabe YC, Nicol MP, Holshouser M, Armstrong DT, et al. Diagnostic accuracy of a rapid urine lipoarabinomannan test for tuberculosis in HIV-infected adults. J Acquir Immune Defic Syndr. 2014;66(3):270-9.
89. Ngangue YR, Mbuli C, Neh A, Nshom E, Koudjou A, Palmer D, et al. Diagnostic Accuracy of the Truenat MTB Plus Assay and Comparison with the Xpert MTB/RIF Assay to Detect Tuberculosis among Hospital Outpatients in Cameroon. J Clin Microbiol. 2022;60(8):e0015522.
90. Nguyen DNT, Nguyen TV, Dao TT, Nguyen LT, Horby P, Nguyen KV, et al. One year experience using mycobacterial blood cultures to diagnose tuberculosis in patients with prolonged fever in Vietnam. Journal of Infection in Developing Countries. 2014;8(12):1620-4.
91. Pandey V, Singh P, Singh S, Arora N, Quadir N, Singh S, et al. SeeTB: A novel alternative to sputum smear microscopy to diagnose tuberculosis in high burden countries. Sci Rep. 2019;9(1):16371.
92. Pant P, Gurung K, Shrestha N, Basnet S. GeneXpert Based Confirmed Cases among Suspected Cases of Tuberculosis in a Tertiary Care Centre: A Descriptive Cross-sectional Study. JNMA J Nepal Med Assoc. 2022;60(247):250-3.
93. Penn-Nicholson A, Gomathi SN, Ugarte-Gil C, Meaza A, Lavu E, Patel P, et al. A prospective multicentre diagnostic accuracy study for the Truenat tuberculosis assays. Eur Respir J. 2021;58(5).
94. Peter JG, Theron G, van Zyl-Smit R, Haripersad A, Mottay L, Kraus S, et al. Diagnostic accuracy of a urine lipoarabinomannan strip-test for TB detection in HIV-infected hospitalised patients. Eur Respir J. 2012;40(5):1211-20.
95. Peter JG, Zijenah LS, Chanda D. Erratum: Effect on mortality of point-of-care, urine-based lipoarabinomannan testing to guide tuberculosis treatment initiation in HIV-positive hospital inpatients: A pragmatic, parallel-group, multicountry, open-label, randomised controlled trial. (Lancet (2016) 387 (1187-97)). The Lancet. 2016;387(10029):1722.
96. Quincó P, Bührer-Sékula S, Brandão W, Monte R, Souza SL, Saraceni V, et al. Increased sensitivity in diagnosis of tuberculosis in HIV-positive patients through the small-membrane-filter method of microscopy. J Clin Microbiol. 2013;51(9):2921-5.
97. Rachow A, Saathoff E, Mindru R, Popescu O, Lugoji D, Mahler B, et al. Diagnostic performance of the AID line probe assay in the detection of Mycobacterium tuberculosis and drug resistance in Romanian patients with presumed TB. PLoS One. 2022;17(8):e0271297.
98. Reddy KP, Brady MF, Gilman RH, Coronel J, Navincopa M, Ticona E, et al. Microscopic observation drug susceptibility assay for tuberculosis screening before isoniazid preventive therapy in HIV-infected persons. Clin Infect Dis. 2010;50(7):988-96.
99. Reddy S, Ntoyanto S, Sakadavan Y, Reddy T, Mahomed S, Dlamini M, et al. Detecting Mycobacterium tuberculosis using the loop-mediated isothermal amplification test in South Africa. Int J Tuberc Lung Dis. 2017;21(10):1154-60.

100. Reeve BWP, Ndlangalavu G, Mishra H, Palmer Z, Tshivhula H, Rockman L, et al. Point-of-care C-reactive protein and Xpert MTB/RIF Ultra for tuberculosis screening and diagnosis in unselected antiretroviral therapy initiators: a prospective diagnostic accuracy study. 2023.
101. Sander MS, Laah SN, Titahong CN, Lele C, Kinge T, de Jong BC, et al. Systematic screening for tuberculosis among hospital outpatients in Cameroon: The role of screening and testing algorithms to improve case detection. Journal of Clinical Tuberculosis and Other Mycobacterial Diseases. 2019;15.
102. Sani FM, Uba A, Tahir F, Abdullahi IN, Adekola HA, Mustapha J, et al. Spectrum of pulmonary fungal pathogens, associated risk factors, and anti-fungal susceptibility pattern among persons with presumptive tuberculosis at Gombe, Nigeria. Int J Mycobacteriol. 2020;9(2):144-9.
103. Santoso P, Soeroto AY, Juniati R, Hartantri Y, Wisaksana R, Alisjabana B, et al. Improving Diagnostic of Pulmonary Tuberculosis in HIV Patients by Bronchoscopy: A Cross Sectional Study. Acta Med Indones. 2017;49(4):330-5.
104. Scott LE, McCarthy K, Gous N, Nduna M, Van Rie A, Sanne I, et al. Comparison of Xpert MTB/RIF with other nucleic acid technologies for diagnosing pulmonary tuberculosis in a high HIV prevalence setting: a prospective study. PLoS Med. 2011;8(7):e1001061.
105. Seong GM, Lee J, Lee JH, Kim JH, Kim M. Usefulness of sputum induction with hypertonic saline in a real clinical practice for bacteriological yields of active pulmonary tuberculosis. Tuberc Respir Dis (Seoul). 2014;76(4):163-8.
106. Shah M, Paradis S, Betz J, Beylis N, Bharadwaj R, Caceres T, et al. Multicenter Study of the Accuracy of the BD MAX Multidrug-resistant Tuberculosis Assay for Detection of Mycobacterium tuberculosis Complex and Mutations Associated With Resistance to Rifampin and Isoniazid. Clin Infect Dis. 2020;71(5):1161-7.
107. Shinu P, Nair A, Jad B, Singh V. Evaluation of two pretreatment methods for the detection of Mycobacterium tuberculosis in suspected pulmonary tuberculosis. J Basic Microbiol. 2013;53(3):260-7.
108. Solari L, Alarcón JO, Piscoya J, Tejada R. [Respiratory symptoms in people attended in health facilities of the Ministry of Health in Lima, Peru]. Rev Peru Med Exp Salud Publica. 2019;36(2):207-13.
109. Songkhla MN, Tantipong H, Tongsai S, Angkasekwinai N. Lateral Flow Urine Lipoarabinomannan Assay for Diagnosis of Active Tuberculosis in Adults With Human Immunodeficiency Virus Infection: A Prospective Cohort Study. Open Forum Infect Dis. 2019;6(4):ofz132.
110. Spooner E, Reddy S, Ntoyanto S, Sakadavan Y, Reddy T, Mahomed S, et al. TB testing in HIV-positive patients prior to antiretroviral treatment. Int J Tuberc Lung Dis. 2022;26(3):224-31.
111. Vadwai V, Shetty A, Rodrigues C. Multiplex allele specific PCR for rapid detection of extensively drug resistant tuberculosis. Tuberculosis (Edinb). 2012;92(3):236-42.
112. van Hoving DJ, Meintjes G, Maartens G, Kengne AP. A multi-parameter diagnostic clinical decision tree for the rapid diagnosis of tuberculosis in HIV-positive patients presenting to an emergency centre. Wellcome Open Res. 2020;5:72.
113. van Lettow M, Bedell R, Maosa S, Phiri K, Chan AK, Mwinjiwa E, et al. Outcomes and Diagnostic Processes in Outpatients with Presumptive Tuberculosis in Zomba District, Malawi. PLoS One. 2015;10(11):e0141414.

114. Vijayageetha M, Kumar AM, Ramakrishnan J, Sarkar S, Papa D, Mehta K, et al. Tuberculosis screening among pregnant women attending a tertiary care hospital in Puducherry, South India: is it worth the effort? Glob Health Action. 2019;12(1):1564488.
115. Wake RM, Govender NP, Omar SV, Ismail F, Tiemessen CT, Harrison TS, et al. Rapid urine-based screening tests increase the yield of same-day tuberculosis diagnoses among patients living with advanced HIV disease. Aids. 2022;36(6):839-44.
116. Wang SF, Ou XC, Li Q, Zheng HW, Wang YF, Zhao YL. The Abbott RealTime MTB assay and the Cepheid GeneXpert assay show comparable performance for the detection of Mycobacterium tuberculosis in sputum specimens. Int J Infect Dis. 2016;45:78-80.
117. Xu P, Tang P, Song H, Zhao J, Chen H, Xue J, et al. The incremental value of bronchoalveolar lavage for the diagnosis of pulmonary tuberculosis in a high-burden urban setting. J Infect. 2019;79(1):24-9.
118. Yeong C, Byrne AL, Cho JG, Sintchenko V, Crighton T, Marais BJ. Use of GeneXpert MTB/RIF on a single pooled sputum specimen to exclude pulmonary tuberculosis among hospital inpatients placed in respiratory isolation. Int J Infect Dis. 2020;92:175-80.
119. Yu X, Wang F, Ren R, Dong L, Xue Y, Zhao L, et al. Xpert MTB/RIF Ultra Assay Using Stool: an Effective Solution for Bacilli Identification from Adult Pulmonary Tuberculosis Suspects without Expectorated Sputum. Microbiol Spectr. 2023;11(4):e0126523.
120. Zhao Z, Wu T, Wang M, Chen X, Liu T, Si Y, et al. A new droplet digital PCR assay: improving detection of paucibacillary smear-negative pulmonary tuberculosis. Int J Infect Dis. 2022;122:820-8.
